# A retrospective analysis of the diagnostic performance of ^11^C-choline PET/CT for detection of hyperfunctioning parathyroid glands after prior negative or discordant imaging in primary hyperparathyroidism

**DOI:** 10.1186/s13550-021-00778-7

**Published:** 2021-03-26

**Authors:** M. E. Noltes, S. Kruijff, L. Jansen, H. E. Westerlaan, W. T. Zandee, R. A. J. O. Dierckx, A. H. Brouwers

**Affiliations:** 1 Department of Nuclear Medicine and Molecular Imaging, University of Groningen, University Medical Center Groningen, Hanzeplein 1, 9700 RB Groningen, The Netherlands; 2 Department of Surgical Oncology, University of Groningen, University Medical Center Groningen, Groningen, The Netherlands; 3 Department of Radiology, University of Groningen, University Medical Center Groningen, Groningen, The Netherlands; 4 Department of Internal Medicine, Division of Endocrinology, University of Groningen, University Medical Center Groningen, Groningen, The Netherlands

**Keywords:** Primary hyperparathyroidism (pHPT), ^11^C-choline PET/CT, Minimally invasive parathyroidectomy (MIP), Endocrine

## Abstract

**Background:**

Identifying the correct location of a parathyroid adenoma in patients with primary hyperparathyroidism (pHPT) is crucial as it can guide surgical treatment. This study aimed to determine the diagnostic performance of ^11^C-choline PET/CT in patients with pHPT as a next in-line scan after primary negative or discordant first-line imaging.

**Methods:**

This was a retrospective single-center cohort study. All patients with pHPT that were scanned utilizing ^11^C-choline PET/CT, after prior negative or discordant imaging, between 2015 and 2019 and who subsequently underwent parathyroid surgery were included. The results of the ^11^C-choline PET/CT were evaluated lesion-based, with surgical exploration and histopathological examination as the gold standard.

**Results:**

In total, 36 patients were included of which three patients were known to have Multiple Endocrine Neoplasia (MEN) syndrome. In these 36 patients, 40 lesions were identified on ^11^C-choline PET/CT and 37 parathyroid lesions were surgically removed. In 34/36 (94%) patients a focused parathyroidectomy was performed, in one patient a cervical exploration due to an ectopically identified adenoma, and in one patient a bilateral exploration was performed because of a double adenoma. Overall, per-lesion sensitivity of ^11^C-choline PET/CT was 97%, the positive predictive value was 95% and the accuracy was 94% for all parathyroid lesions.

**Conclusions:**

In patients with pHPT and prior negative or discordant first-line imaging results, pathological parathyroid glands can be localized by ^11^C-choline PET/CT with high sensitivity and accuracy.

## Background

Primary hyperparathyroidism (pHPT) is the most common cause of hypercalcemia, mainly occurring in postmenopausal women [1]. The leading cause of pHPT is a solitary adenoma (89%) followed by multiple gland hyperplasia (6%), double adenomas (4%) and parathyroid carcinomas (< 1%) [2].

The treatment of pHPT is generally surgical removal of the hyperfunctioning parathyroid gland(s). Since a solitary adenoma is the predominant cause, parathyroid surgery is preferably performed through a minimally invasive parathyroidectomy (MIP) in which only the suspected adenoma is resected in a focused manner. The other classic surgical approach is a bilateral neck exploration (BNE), in which all four parathyroid glands are exposed and inspected, and subsequently the enlarged glands are resected.

Accurate preoperative imaging is pivotal to facilitate a MIP. Widely used first-line imaging modalities include ^99m^Tc-methoxyisobutylisonitrile-single-photon emission computed tomography/(computed tomography) (^99m^Tc-MIBI-SPECT/(CT)) and cervical ultrasonography (cUS). The combined use of these scans can reach a sensitivity of 80–90% [3–5]. For the remaining 10–20% of patients, a BNE could be avoided if other innovative functional imaging techniques using positron emission tomography (PET)/CT, with radiotracers such as ^11^C-methionine and ^11^C-choline/^18^F-fluorocholine, would be able to localize the adenoma [6–10].

The sensitivity of ^11^C-methionine PET/CT is reported 72–86% [6, 7, 11, 12], whereas in ^18^F-fluorocholine PET/CT sensitivity is 90–96% [8–10, 13–15]. Studies regarding the diagnostic performance of ^11^C-choline PET/CT are however scarce. Only three studies have investigated the accuracy of ^11^C-choline PET/CT showing a sensitivity of 87%, 92% and 99%, of which two studies also included patients with positive ^99m^Tc-MIBI scans [16–18]. Furthermore, we have recently optimized the imaging protocol of ^11^C-choline PET/CT for the detection of parathyroid adenomas [19].

Therefore, this study aimed to analyze the diagnostic performance of ^11^C-choline PET/CT after prior negative or discordant first-line imaging in patients with pHPT undergoing parathyroid surgery with an optimized imaging protocol.

## Methods

### Study design and patient selection

This is a retrospective single-center cohort study of patients ≥ 18 years with biochemically proven pHPT who underwent parathyroid surgery after localization utilizing ^11^C-choline PET/CT and negative or discordant first-line imaging in a teaching and tertiary referral hospital in the Netherlands between 2015 and 2019. Patients with a germline mutation predisposing for familial hypocalciuric hypercalcemia were excluded. First-line imaging results were classified as discordant if a potential adenoma was identified on only one imaging modality or both imaging modalities showed an adenoma but in different locations.

The medical charts were reviewed to determine the outcome of all preoperative imaging tests. The location was recorded from imaging reports. Localization of suspected parathyroid adenoma(s) was described in relation to the position of the thyroid midline/trachea, i.e. right cranial; right caudal; left cranial; left caudal; ectopic (e.g. paratracheoesophageal or mediastinal) or no parathyroid adenoma identified. Corrected serum calcium levels were calculated [20].

A no objection notice of subjects was obtained after the protocol had been approved by the Medical Ethics Committee UMC Groningen (2018/658).

### Cervical ultrasonography

cUS was performed in the University Medical Center Groningen (UMCG) or referring hospitals on various ultrasound systems by radiologists, as described previously [6]. In short; patients were examined in a supine position with a hyperextended neck using a high-frequency linear transducer. The neck was scanned from the level above the thyroid to the clavicle caudally. In case the cUS was performed outside the UMCG, images were retrieved, however re-interpretation was not performed, due to the highly operator dependent nature of this imaging modality.

### ^99m^Tc-MIBI-SPECT(/CT)

At the UMCG patients were scanned on a Symbia T16 gamma camera with CT (Siemens), resulting in SPECT/CT images. ^99m^Tc-MIBI was used for preoperative localization as dual phase technique, which was combined with a dual tracer subtraction technique for thyroid only visualization with ^99m^Tc-pertechnetate. Some ^99m^Tc-MIBI scans were performed in referring hospitals with slightly different imaging protocols (only dual phase technique and/or only SPECT). However, all protocols adhered to the international guidelines on parathyroid imaging [21]. The ^99m^Tc-MIBI-SPECT(/CT) were re-interpreted by UMCG nuclear medicine physicians in case it had been performed outside the UMCG.

### ^11^C-choline PET/CT

^11^C-choline PET/CT was performed as described previously by Noltes et al. [19]. In short; patients had to fast for six hours while drinking one liter of water, tea or coffee without milk and sugar prior to the PET/CT procedure. First, a low dose CT (ldCT) was performed for attenuation correction of the PET images, with 120 kV, Quel ref mAs of 30 and a pitch of 1.5 on a 40 or 64-slice CT (Biograph mCT, Siemens). Scan area involved one bed position, was from the lower jaw to the heart and was recorded in listmode. In the first five patients, PET/CT images were taken directly after injection of a median dose of 556 MBq (range 510–635 MBq) ^11^C-choline until 40 to 60 min postinjection. After optimizing our imaging protocol [19], all subsequent patients were scanned dynamically 20 min after injection of a median dose of 410 MBq (range 360–440 MBq) for 10 min. All images were iteratively reconstructed using three iterations, 21 subsets with 5 mm FWHM Gaussian filter, including time of flight and resolution modelling. A lesion was called positive and suspected to be a parathyroid adenoma in case there was focal uptake above background, and the location could match with a parathyroid location.

### Surgery

Parathyroidectomy was performed by experienced surgeons either via a MIP or a BNE. Intraoperative parathyroid hormone (ioPTH) was measured at T0 (before incision), T1 (excision of adenoma), T2 (T1 + 5 min), T3 (T1 + 10 min) and T4 (T1 + 15 min). A decrease of ioPTH of ≥ 50%[22] was classified as sufficient. Final localization of the adenoma during surgery was retrieved from the surgical report. The pathology report was reviewed for the final diagnosis. Cure was defined as normal serum calcium levels (< 2.57 mmol/L) 6 months after surgery [23].

Lesion based localization was defined as true positive, true negative, false positive, and false negative, depending on the final outcome (i.e. surgical and pathology reports). A true positive scan was defined as suspected adenoma(s) localized to the correct quadrant. A scan was considered true negative when no pathological parathyroid gland(s) was found at surgery and the scan suggested no presence of hyperactive parathyroid tissue. A false positive scan was defined as suspected adenoma(s) localized to the incorrect quadrant or a positive scan in a quadrant while during surgery no pathological parathyroid gland was found in this quadrant. A false negative scan was defined as removal of a pathological parathyroid gland during surgery, although the scan had not demonstrated the presence of hyperactive parathyroid tissue in this quadrant.

Per-lesion sensitivity, PPV and accuracy (of intraoperatively inspected parathyroid glands) of ^11^C–choline PET/CT was calculated.

### Statistics

Data were analyzed using descriptive statistics. Categorical variables are displayed as count (n) and percentage (%). Continuous variables are displayed by median with range. Statistical analyses were performed using IBM SPSS Statistics version 25.0 (IBM Corp., Armonk, NY, USA).

## Results

### Patients

**Fig. 1:**
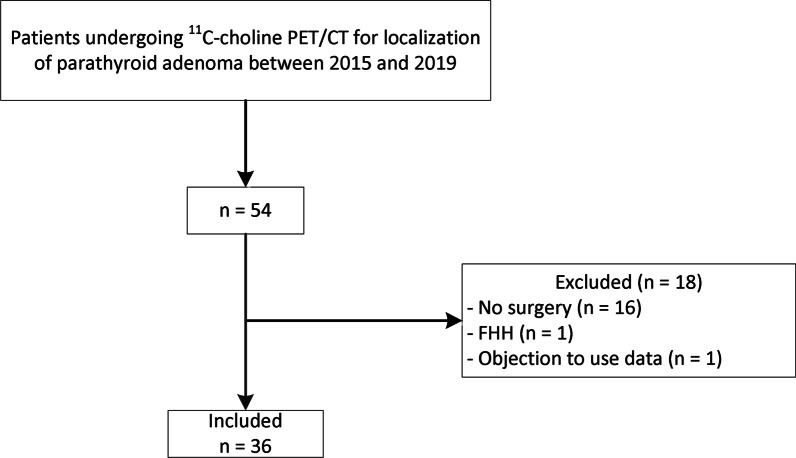
Flowchart of patient inclusion. *FHH* = familial hypocalciuric hypercalcemia

Between 2015 and 2019, 54 patients underwent a ^11^C-choline PET/CT. After exclusion of 18 patients, 36 patients were included in this study (Fig. 1). Data from 13 patients were previously published [24].

Thirty patients were female (83%), the median age was 64 (36–76) years and median preoperative corrected calcium and PTH levels were 2.78 (2.42–3.62) mmol/L and 15.0 (7.4–125.0) pmol/L, respectively (Table 1). Nine patients had a previous parathyroidectomy (four positive and five negative explorations), and three patients were diagnosed with Multiple Endocrine Neoplasia (MEN) syndrome preoperatively (two with MEN-I and one with MEN-IIA).

**Table 1:** Patients’ baseline characteristics

Characteristics	Total (n = 36)
Gender, n (%)	
Female	30 (83%)
Age, years	
Median (range)	64 (36–76)
Body mass index, kg/m^2^	
Median (range)	27.4 (18.5–40.2)
Preoperative total serum calcium (mmol/L)	
Median (range)	2.87 (2.58–3.58)
Preoperative albumin-corrected calcium (mmol/L)	
Median (range)	2.78 (2.42–3.62)
Preoperative PTH (pmol/L)	
Median (range)	15.0 (7.4–125.0)
Previous parathyroidectomy, n (%)	
Yes	9 (25%)

Reference range total serum calcium = 2.20–2.60 mmol/L; reference range albumin-corrected calcium = 2.10–2.55 mmol/L; reference range PTH =< 8.7 pmol/L

### Preoperative imaging

A cUS was performed in 30/36 patients (24 at the UMCG and six at other hospitals), of which in nine patients a parathyroid adenoma could not be identified (Table 2). In the remaining 21 patients, an adenoma was suspected on cUS, but this lesion could not be confirmed on ^99m^Tc-MIBI-SPECT(/CT) (discordant findings n = 16) or a ^99m^Tc-MIBI-SPECT(/CT) was not performed (n = 5). A ^99m^Tc-MIBI-SPECT(/CT) was performed in 29 of the 36 patients (20 at the UMCG and nine at other hospitals). ^99m^Tc-MIBI-SPECT(/CT) was negative in 21 patients and positive but discordant with cUS in eight patients (Table 2).

**Table 2:** Preoperative localization results of ultrasonography and ^99m^Tc-MIBI-SPECT(/CT) of 36 patients

Ultrasound	^99m^Tc-MIBI-SPECT(/CT)		Not performed	Total
	Positive	Negative		
Positive	3*	13	5	21
Negative	5	2	2	9
Not performed	0	6	NA	6
**Total**	8	21	7	36

*Positive for both imaging modalities, but discordant location; NA = not applicable

**Fig. 2:**
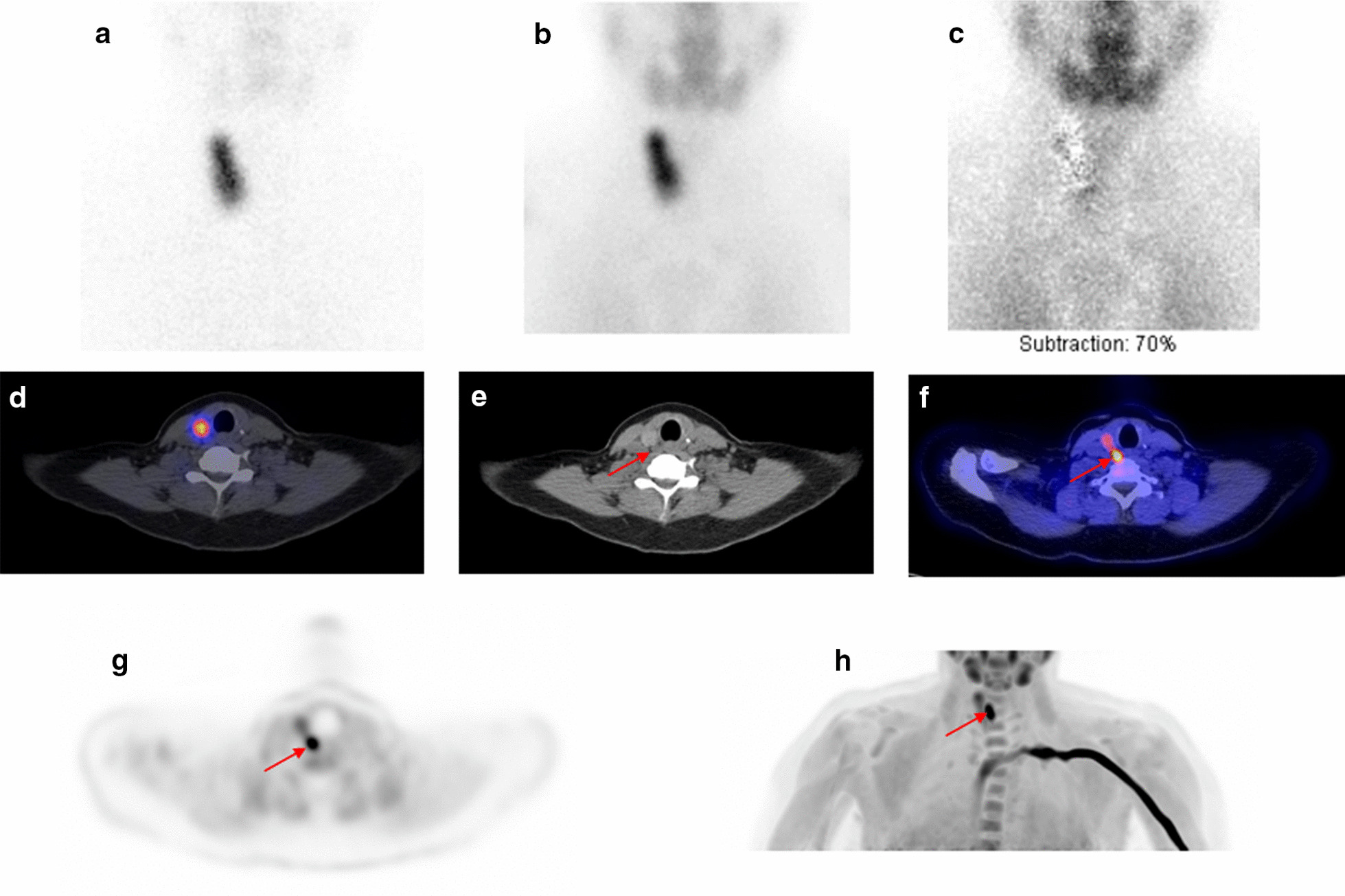
Patient example of a negative ^99m^Tc-MIBI-SPECT/CT and a positive ^11^C-choline PET/CT. Previously, the patient underwent a hemithyroidectomy on the left side because of a thyroid nodule, which proved to be benign. Planar anterior image of the neck with ^99m^Tc-pertechnetate (**a**) and early ^99m^Tc-MIBI (**b**) showing physiological uptake in the right thyroid lobe. Planar subtraction image (early ^99m^Tc-MIBI minus ^99m^Tc-pertechnetate image **c**) also does not show the parathyroid adenoma. The uptake in the right thyroid lobe was also observed on the ^99m^Tc-MIBI-SPECT image **d**. The low dose-CT of the ^99m^Tc-MIBI-SPECT/CT **e** retrospectively did show a focus suspect for a parathyroid adenoma located paraoesophageally on the right side (red arrow). The ^11^C-choline PET/CT (**f** fused PET/CT image and **g**, **h** PET only image) showed this same lesion (1.80 cm and 1.00 g at pathology) located paraoesophageally on the right side, suspicious for a parathyroid adenoma (red arrow). The ^11^C-choline PET/CT also showed the same physiological uptake in the right thyroid lobe (**f**–**h**)

^11^C-choline PET/CT was positive in all 36 patients, and 40 lesions suspicious for a parathyroid adenoma were identified on ^11^C-choline PET/CT. Figure 2 shows representative images of a patient with a negative ^99m^Tc-MIBI-SPECT(/CT) and a positive ^11^C-choline PET/CT. Of the excluded 16 patients who did not undergo surgery, in four patients (25%) a parathyroid lesion could not be identified on ^11^C-choline PET/CT.

### Surgery

In 34/36 patients (94%) a unilateral parathyroidectomy was performed. In one patient with an ectopically identified adenoma on ^11^C-choline PET/CT, the adenoma was extirpated from the mediastinum through a cervical incision. In the second patient, a BNE was performed because of a right and left-sided identified parathyroid lesion. In 33 patients, ioPTH was used and showed a sufficient decrease in 32 patients. The patient without a sufficient decrease was diagnosed with postoperative mildly persisting HPT.

In total, of the 40 suspected lesions, 37 were histologically confirmed as parathyroid adenoma (n = 33) or parathyroid hyperplasia (n = 4). Median weight and diameter of sporadic parathyroid adenomas was 0.39 (0.10–13.40) gram and 1.5 (0.50–6.00) cm and for MEN related parathyroid lesions 0.90 (0.08–36.00) gram and 1.5 (0.90–1.50) cm, respectively.

In one patient with MEN-I syndrome, ^11^C-choline PET/CT identified two parathyroid adenomas, but only one adenoma was resected (true positive). Since this MEN-I patient already had two parathyroid glands resected, the decision was made to perform a MIP and leave one parathyroid gland in-situ to avoid hypoparathyroidism. In one patient with sporadic pHPT, ^11^C-choline PET/CT identified three possible parathyroid adenomas on both sides of the neck, but during surgery only the right side was explored. Since a sufficient decrease of ioPTH was found after removal of one parathyroid adenoma, the decision was made not to explore the left side, resulting in one true positive and one false positive lesion. Thus, in these two patients, two lesions on ^11^C-choline PET/CT were not confirmed as parathyroid adenomas and were excluded, leaving 38 ^11^C-choline PET/CT lesions for analysis.

^11^C-choline PET/CT was true positive in 36 lesions (34 sporadic, two MEN-I lesions), false positive in two (one sporadic, one MEN-IIA lesion), false negative in one lesion (MEN-IIA) and true negative in eight lesions (all sporadic) (Table 3). In eight patients, the parathyroid gland on the ipsilateral site was explored as well, showing normal glands (true negative). The two false positive and the one false negative cases were analyzed (Table 4).

**Table 3:** Final ^11^C-choline PET/CT lesion-based outcome for the 37 resected adenomas

^11^C-choline PET/CT	Surgery + pathology sporadic pHPT lesions		Surgery + pathology MEN-related lesions	
	Positive	Negative	Positive	Negative
Positive	34	1	2	1
Negative	0	8	1	0

pHPT = primary hyperparathyroidism; MEN = multiple endocrine neoplasia

**Table 4:** Outcomes of the false positive (n = 2) and false negative (n = 1) ^11^C-choline PET/CT in n = 2 patients

Clinical diagnosis	pHPT	MEN-IIA
cUS	RL, LL	RL
^99m^Tc-MIBI-SPECT(/CT)	Ectopic	Negative
^11^C-choline PET/CT	RL, RU, LU	RL
Surgical technique	Minimal invasive	Minimal invasive
Intra-operative localization	RL	RU
ioPTH	> 50% decrease	NA
Pathology	Adenoma, lymph node, thyroiditis	Hyperplasia
Follow-up	Cured	Cured
^11^C-choline PET/CT result	TP, FP, one lesion not confirmed	FP, FN

*cUS* = cervical ultrasonography; *pHPT* = primary hyperparathyroidism; *ioPTH* = intraoperative PTH; *RL* = right lower quadrant; *LL* = left lower quadrant; *RU* = right upper quadrant; *LU* = left upper quadrant; *TP* = true positive; *FP* = false positive; *MEN* = Multiple Endocrine Neoplasia; *NA* = not available; *FN* = false negative

There was one patient with MEN-IIA syndrome where the ^11^C-choline PET/CT showed a lesion in the right lower quadrant, but the adenoma was localized elsewhere during surgery (right upper). Hence, a false positive and false negative localization was registered (Table 4). This false negative lesion could in retrospect be identified on ^11^C-choline PET/CT. The other false positive lesion includes the aforementioned patient with adenomas on both sides of the neck. In retrospect, the false positive lesions may have been due to reactive lymph nodes and thyroiditis, as was found in the pathological examination (Table 4).

Overall, per-lesion sensitivity of ^11^C-choline PET/CT for all parathyroid lesions was 97% (100% for sporadic lesions and 67% for MEN related lesions, respectively), the PPV 95% (97% sporadic and 67% MEN) and the accuracy 94% (98% sporadic and 50% MEN).

### Follow-up

Median albumin-corrected calcium and PTH level on postoperative day one and six months post-surgery were 2.37 (2.00–2.75) mmol/L and 2.2 (1.0–5.2) pmol/L, and 2.37 (2.10–2.69) mmol/L and 9.9 (1.2–18.8) pmol/L, respectively (Table 5). In total, 34/36 patients were cured two months post-surgery and 10/12 patients (24 loss to follow up) 6 months post-surgery. One patient that was not cured was diagnosed with MEN-I and during surgery, the decision was made to leave one parathyroid gland in-situ to avoid hypoparathyroidism. The other patient presented with mildly persisting HPT. Additional imaging did not show a definitive localization and due to the mildly elevated albumin-corrected calcium levels (2.61 mmol/L) no further surgical or medical intervention was deemed necessary.

**Table 5:** Calcium and PTH levels post-surgery

	Postoperative day one	Two months post-surgery	Six months post-surgery
Total serum calcium (mmol/L)			
Median (range)	2.35 (2.00–2.73) (n = 36)	2.40 (2.18–2.67) (n = 14)	2.41 (2.03–2.83) (n = 12)
Albumin-corrected calcium (mmol/L)			
Median (range)	2.37 (2.00–2.75) (n = 34)	2.37 (2.08–2.60) (n = 14)	2.37 (2.10–2.69) (n = 12)
PTH (pmol/L)			
Median (range)	2.2 (1.0–5.2) (n = 14)	8.6 (1.2–18.8) (n = 8)	9.9 (1.2–18.8) (n = 7)

Reference range total serum calcium = 2.20–2.60 mmol/L; reference range albumin-corrected calcium = 2.10–2.55 mmol/L; reference range PTH =  < 8.7 pmol/L

## Discussion

In this study, ^11^C-choline PET/CT showed overall a high lesion-based sensitivity, PPV and accuracy of 97%, 95% and 94%, respectively in 36 patients operated for pHPT with prior non-conclusive first-line imaging. The surgeon performed a successful resection in all 36 patients of which in 34 patients a MIP was sufficient and an extensive neck exploration was avoided.

The overall sensitivity of 97% in our study is comparable to three other studies, showing a sensitivity of 87.0%, 92.3% and 98.8% [16–18]. Our sensitivity would have been even higher if we had excluded MEN-IIA patients from our analysis (100% for sporadic pHPT and 100% for MEN-I patients). Liu et al*.* conducted a study of 87 patients undergoing ^11^C-choline PET/CT after negative or discordant first-line imaging and subsequently parathyroid surgery [18]. They defined lesions with both positive and inconclusive uptake as positive ^11^C-choline PET lesions, which might have overestimated their diagnostic accuracy. Two other studies both included patients with pHPT undergoing ^11^C-choline PET/CT and parathyroid surgery in whom the adenoma was also detected on MIBI imaging [16, 17]. The main strength of our study is that it describes and reflects the accuracy of ^11^C-choline PET/CT after prior negative or discordant first-line imaging in daily practice.

We included MEN-I and MEN-IIA patients since preoperative imaging is pivotal in both MEN-I patients with recurrent disease and MEN-IIA patients [23]. This patient group was, however, limited in our cohort and more research is warranted into additional preoperative imaging in MEN patients.

The use of a highly sensitive preoperative imaging technique, such as ^11^C-choline PET/CT might lead to more minimally invasive procedures. Correspondingly, the surgeon performed a successful MIP in 34/36 patients. A MIP has several advantages compared to a BNE. There is a lower risk of postoperative hypocalcemia and laryngeal nerve injury, it results in less unnecessary scarring with better opportunity for later surgery and has a shorter duration of surgery [25, 26]. It is, however, not recommended to employ ^11^C-choline PET/CT as a first-in-line imaging technique since the AAES guideline advises initial imaging with cUS and ^99m^Tc-MIBI-SPECT/CT (or four dimensional-CT), because this is the most cost-effective strategy [23]. If these first-line imaging results are negative, imaging upscaling can be performed, e.g. with ^11^C-choline PET/CT. More research into the cost-effectiveness of ^11^C-choline PET/CT is warranted to study its feasibility as a first-line imaging technique.

The studied imaging technique, ^11^C-choline PET/CT, has potential drawbacks, such as that the tracer production of ^11^C-choline is complicated and expensive, includes the need for a cyclotron on site because of its short half-life (20 min) and is therefore restricted in its availability. Therefore, other PET imaging techniques for the detection of parathyroid lesions have been evaluated. ^18^F-fluorocholine, a tracer comparable to ^11^C-choline, is a more widely available tracer because of its stable biodistribution and longer half-life (110 min). It, therefore, does not require a cyclotron on site. However, biodistributions of these tracers may not be completely identical. This will remain an open question since a direct comparison between the two choline tracers will not likely be performed. Thus far, the reported sensitivity of ^11^C-choline for the detection of parathyroid adenomas is in the range of 92–99%, and studies utilizing ^18^F-fluorocholine report 90–96%, indicating a near-similar performance [8–10, 16, 18]. We estimate a doubling of radiation exposure for ^18^F-fluorocholine compared to ^11^C-choline based on differences in half-life time, positron-energy and biological processes such as metabolism and excretion [27]. Therefore, for our clinical setting, ^11^C-choline seems to be the most feasible. Another feasible option for the detection of parathyroid lesions is ^11^C-methionine PET/CT or four dimensional-CT, with a sensitivity of 72–86% and 80–90%, respectively [6, 7, 11, 12, 28–30]. In our center, we found a sensitivity of 72% for ^11^C-methionine PET/CT in the same clinical setting as the current study [6]. A head-to-head comparison, including a cost-effectiveness analysis, is needed to assess which of these imaging techniques is superior.

Our study has known limitations for retrospective analyses. First, this study was subjected to a referral bias since we only included patients who underwent ^11^C-choline PET/CT and subsequent parathyroid surgery. However, we feel that referral bias was not substantial since in 75% of patients not undergoing surgery, a possible location for a parathyroid lesion was identified on ^11^C-choline PET/CT. Assuming that in the non-operated patients ^11^C-choline PET/CT was correctly positive in 12 patients (75%) and false negative in four patients (25%), this would still have resulted in a high sensitivity of 90% (47/52) for this technique patient-based*.* Second, due to the retrospective nature, there are missing values in the follow-up. Most patients were lost to follow-up two months postoperatively. Thus, we cannot make firm conclusions about the final cure rate of this patient cohort. However, this was not the aim of our retrospective analysis since our primary objective was to correlate preoperative ^11^C-choline PET with surgical localization of parathyroid glands and pathology outcome. Another potential drawback is that not all parathyroid glands were routinely inspected during surgery. This was only the case in 8/36 patients. Some positive lesions were therefore not confirmed as true or false positive, which might also have overestimated the sensitivity of ^11^C-choline PET/CT.

## Conclusion

In patients with pHPT and prior negative or discordant first-line imaging results, pathological parathyroid glands can be localized preoperatively with high accuracy by ^11^C-choline PET/CT. In this challenging clinical setting, ^11^C-choline PET/CT showed a high sensitivity, PPV and accuracy of 97%, 95% and 94%, respectively. The use of a highly sensitive preoperative imaging technique such as ^11^C-choline PET/CT leads to more focused surgical procedures instead of bilateral neck explorations. Further studies are needed, including a head-to-head comparison and cost-effectiveness analysis on the various anatomical and nuclear imaging techniques to determine the order in which to use them in the setting of pHPT.

## Data Availability

The datasets generated and/or analysed during the current study are available from the corresponding author on reasonable request.

## Abbreviations

AAES: American Association of Endocrine Surgeons
CT: Computed tomorgraphy
cUS: Cervical ultrasonography
ioPTH: Intraoperative-PTH
MEN: Multiple Endocrine Neoplasia
^99m^Tc-MIBI-SPECT: ^99M^Tc-methoxyisobutylisonitrile single photon emission computed tomography
PET: Positron emission tomography
pHPT: Primary hyperparathyroidism
PPV: Positive predictive value
PTH: Parathyroid hormone
UMCG: University Medical Center Groningen
